# Flexible Tactile Electronic Skin Sensor with 3D Force Detection Based on Porous CNTs/PDMS Nanocomposites

**DOI:** 10.1007/s40820-019-0288-7

**Published:** 2019-07-16

**Authors:** Xuguang Sun, Jianhai Sun, Tong Li, Shuaikang Zheng, Chunkai Wang, Wenshuo Tan, Jingong Zhang, Chang Liu, Tianjun Ma, Zhimei Qi, Chunxiu Liu, Ning Xue

**Affiliations:** 1 State Key Laboratory of Transducer Technology, Institute of Electronics Chinese Academy of Sciences (IECAS), Beijing, 100190 People’s Republic of China; 2 School of Electronic, Electrical, and Communication Engineering, University of Chinese Academy of Sciences (UCAS), Beijing, 100049 People’s Republic of China; 3 School of Microelectronics, University of Science and Technology of China (USTC), Hefei, 230026 People’s Republic of China

**Keywords:** Flexible tactile sensors, Electronic skin, Piezoresistive sensors, CNTs/PDMS nanocomposites, 3D force detection

## Abstract

Flexible tactile sensors have broad applications in human physiological monitoring, robotic operation and human–machine interaction. However, the research of wearable and flexible tactile sensors with high sensitivity, wide sensing range and ability to detect three-dimensional (3D) force is still very challenging. Herein, a flexible tactile electronic skin sensor based on carbon nanotubes (CNTs)/polydimethylsiloxane (PDMS) nanocomposites is presented for 3D contact force detection. The 3D forces were acquired from combination of four specially designed cells in a sensing element. Contributed from the double-sided rough porous structure and specific surface morphology of nanocomposites, the piezoresistive sensor possesses high sensitivity of 12.1 kPa^−1^ within the range of 600 Pa and 0.68 kPa^−1^ in the regime exceeding 1 kPa for normal pressure, as well as 59.9 N^−1^ in the scope of < 0.05 N and > 2.3 N^−1^ in the region of < 0.6 N for tangential force with ultra-low response time of 3.1 ms. In addition, multi-functional detection in human body monitoring was employed with single sensing cell and the sensor array was integrated into a robotic arm for objects grasping control, indicating the capacities in intelligent robot applications.

**Electronic supplementary material:**

The online version of this article (10.1007/s40820-019-0288-7) contains supplementary material, which is available to authorized users.

## Highlights

- Flexible sensitive carbon nanotubes/polydimethylsiloxane (CNTs/PDMS) nanocomposite with novel double-side rough porous structure was proposed by simple manufacturing methods.
- Three-dimensional (3D) force tactile electronic skin sensor based on CNTs/PDMS nanocompositions exhibited high sensitivity, good consistency and fast response.
- A promising strategy for low-cost multi-functional detection in human body monitoring and intelligent robot grasping applications was provided.

**Electronic supplementary material:**

The online version of this article (10.1007/s40820-019-0288-7) contains supplementary material, which is available to authorized users.

## Graphical abstract

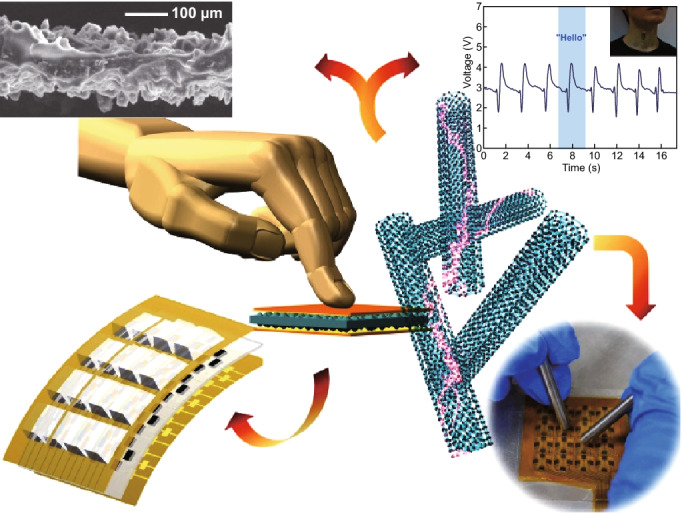

## Introduction

Over past years, wearable and flexible tactile sensors have attracted a great deal of studies due to their great potential in various applications including physiological measurement [1–3], robotics [4], human–computer interaction [5, 6] and wearable devices [7, 8]. Generally speaking, tactile sensors imitate human perception of pressure with capability of detecting shapes and sliding conditions of the contact objects. Compared with silicon-based devices with high hardness and effective Young’s modulus, flexible materials are more suitable for bionic tactile applications due to the good adherence, tensility and flexibility [9–11]. A variety of flexible materials such as polyethylene (PE) [12], polydimethylsiloxane (PDMS) [13, 14], polyurethane (PU) [15] and polyimide (PI) [16] have been applied to tactile sensors, and the operational principles of tactile sensors mainly include piezoresistive [17–19], capacitive [20], piezoelectric [21] and optical [22]. Among them, piezoresistive tactile sensors have been widely used, benefited from their uncomplicated and reliable fabrication process, low cost and application prospects in large area.

Currently, large quantities of conductive nanomaterials and nanocomposites have been demonstrated with piezoresistance such as carbon nanotubes (CNTs) [23, 24], carbon black [25], graphene [26–28], nanowires [12, 29] and metallic particles [5]. Among them, conductive CNTs/polymer composites can realize good piezoresistive effect because the super-high aspect ratio as well as good axial conductivity of CNTs can greatly reduce the material consumption resulting in little change in polymer’s mechanical properties. In addition, CNTs have high mechanical strength and can keep stable properties under repeated external force [30, 31], which makes CNTs a common conductive material in composites [32], microelectronics [33], energy storage and biomedical [34–36]. In terms of sensors and transducers, CNTs are most commonly used in the form of three-dimensional conductive network in both composites and structures composed of pure CNTs. What is more, compared with the complex and vulnerable manufacturing methods of pure CNTs network structure [37], nanocomposites formed by combining CNTs with polymers tend to have a simpler, more stable and lower cost fabrication process and exhibits good stability, conductivity and repeatability at the same time.

Apart from the selection of conducting materials and nanocomposites, the structure of piezoresistive sensing element is also an important factor determining the performance of sensors because different structures possess different moduli of elasticity resulting in different sensitivity and other physical characteristics in practical applications. At present, many microstructures have been used to realize high sensitivity including porous structure [38], pyramid structure [39, 40], micro-pillar structure [41], sponge structure [26], electrospinning structure [12], microdome [42] and hollow cylindrical structure [43]. Sensors with these above-mentioned structures have either high-cost or complex manufacturing process. Moreover, actual tactile contact forces in daily life are usually three-dimensional (3D) with lateral force and sliding, but most present electronic skin devices can only detect external force in the form of normal pressure without tangential force, which limits perception of contact information. Wang et al. [44] and Park et al. [45] have proposed tactile sensors with flexible and sensitive detection for electronic skin applications, but the devices with complex fabrication process cannot effectively detect three-dimensional contact force. Yeo et al. [46] and Buscher et al. [47] have reported wearable pressure sensors with ability of large-area application, but the pressure resolution and sensitivity of the devices are not high enough for tactile sensing. Besides, the proposed 3D force detection sensors still have some shortcomings in tactile sensing. Viry et al. [48] introduced a flexible capacitive three-axial force sensor made with conductive fabric electrodes. The sensor showed sensitive response to pressure but high spatial resolution is hard to realize because of the large volume and the gap between electrodes is susceptible to interference. Lee et al. [49] reported a capacitive tactile sensor array using flexible material for normal and shear force detection. The sensor has good uniformity but small detection range and low sensitivity. The stretchable tri-axial tactile sensor reported by Noda et al. [50] has the same drawbacks of insensitivity and large volume. Therefore, a flexible tactile sensor with the capacity of detecting 3D contact forces sensitively is necessary and promising for bionic and multi-scene applications.

This work demonstrates a 4 × 4 flexible tactile electronic skin sensor based on multi-walled carbon nanotubes (CNTs)/PDMS polymer nanocomposite for 3D contact force detection. The sensor array has good uniformity, spatial resolution and fast response to tiny contact force. High sensitivity in three-axial detection and compatibility with curved surfaces are achieved by using nanocomposite with double-sided rough porous structure and flexible printed circuit (FPC) electrode layers with PI as substrate. The simple preparation and fabrication process of nanocomposite and the device is a great advantage for producing electronic skins in large scale and low cost. Systematic experiments have been done to test the performance of the sensor in specific applications. In addition, the piezoresistive nanocomposite-based pressure sensor has also be applied to measure human wrist pulse, finger bending, limbs movements and robotic object grasping in combination with manipulators, reflecting its potential in applications of human monitoring and robotics.

## Experimental

### Structure and Layout

**Fig. 1:**
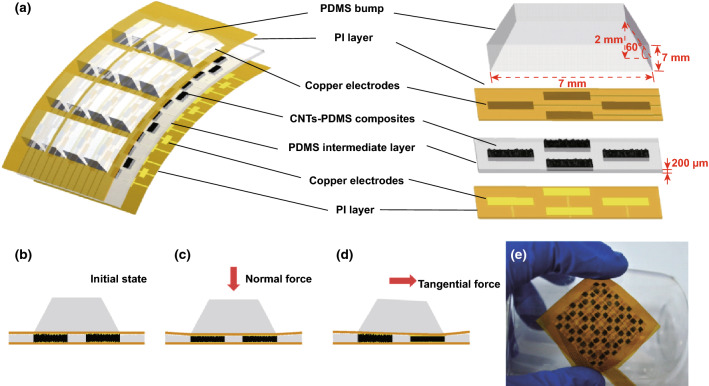
**a** Dismantling structure schematic diagram of the tactile sensor array and one single element. **b**–**d** Cross-sectional diagrams of a tactile sensor element under initial state, normal force state and tangential force state, respectively. **e** Photograph of the sensor array attached to a curved surface

The proposed sensor array was constructed in a sandwich-like structure in which a double-sided rough porous surface structural CNTs/PDMS nanocomposite is sandwiched in between flexible upper and lower electrode layers. Figure 1a schematically depicts the structure of the sensor array (4 × 4 cm^2^) and a single element. The CNTs/PDMS nanocomposites (2 × 2 mm^2^) are sandwiched between two copper electrodes with the same size, and a transparent PDMS intermediate layer with the similar surface morphology and thickness (200 μm) as nanocomposites is used to immobilize nanocomposites. The PDMS intermediate layer as isolating layer is adopted to prop electrode layers up and guarantee the same strain occurring as nanocomposites under pressure loading. The four nanocomposite cells beneath the bump are arranged in middle of four edges of the bump, respectively, and all cells on the same rows and columns in different elements are connected together through the upper electrode and lower electrode, respectively, through which the row-column scanning configuration simplifies the wiring layout of electrode layers and circuit interface. PDMS bumps with 2 mm height and 7 mm bottom side length are used to protect the tactile sensor and transmit 3D force to cells. Figure 1b–d illustrates cross-sectional diagrams of a sensor element under three different loading states which are initial state, normal force (*z* axis) loading state and tangential force (*x* or *y* axis) loading state, respectively. When the tactile sensor is in the initial state without external force loading, the piezoresistive nanocomposites keep multi-points contact with both electrodes surfaces and the gaps between CNTs in nanocomposites remain the maximum. As a normal force is applied to the bump, four cells beneath the bump are subjected to the same pressure and the contact areas between the surfaces of nanocomposites and the electrodes increase, while the gap among CNTs decreases forming a denser conducting network which reduces resistances of piezoresistive nanocomposites in four cells to the same magnitude. As a tangential force is applied, the tilt and deformation of bump will induce the right cell to be subject to undergoing compressive stress while the opposite cell is subjected to tensile stress (Fig. 1d). As a result, the resistance of the former cell reduces while the latter one’s resistance is not significantly changed. The tangential force can be detected by calculating the difference between resistances of two cells in the corresponding axial direction. The sensor composed of fully flexible materials can conform to curved surfaces with the curvature radius of 30 mm as shown in Fig. 1e. What is more, an array scanning circuit is employed in our work for voltage signals acquisition of each piezoresistive cell (Figure S1) and the resistance also can be obtained by proportionate calculation in real time (Supporting Information).

### Fabrication and Working Principle

**Fig. 2:**
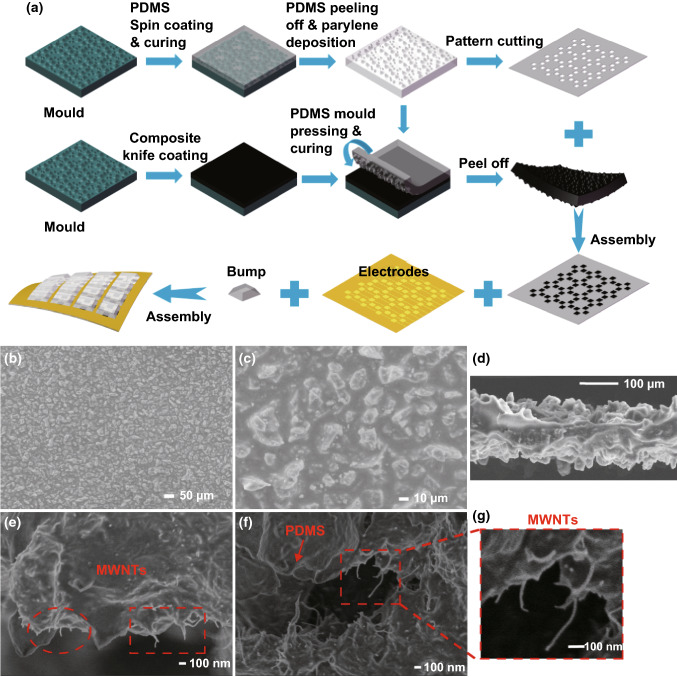
**a** Schematic of the fabrication details of flexible tactile sensor array. **b**, **c** SEM images in top view of surface structure of nanocomposites at different magnifications. **d** Side view of the nanocomposite film with double-sided rough surface. **e**, **f** SEM images of porous structures in the nanocomposite at high magnification. **g** Magnified view of individual CNTs in nanocomposite

Fabrication flow of the tactile sensor array is illustrated in Fig. 2a. Abrasive paper (grit degree: P800) with rough and uneven surface is utilized in our work as mold for flexible materials manufacturing. Firstly, a 1-μm-thick layer of parylene-C is deposited on the rough surface of the abrasive paper mold followed by spin coating and curing of PDMS, then the cured PDMS film is peeled off from the abrasive paper and deposited with 1-μm-thick parylene-C on the rough surface. Secondly, the PDMS film with rough surface is cut into the pattern of the intermediate layer by position stamping. Thirdly, the prepared CNTs/PDMS nanocomposite is knife-coated onto the aforementioned mold and then the flexible PDMS film deposited with parylene-C is covered on the surface of the nanocomposite with the rough surface downward. After heating at 120 °C for 30 min in vacuum, the nanocomposite film possesses double-sided rough surface morphology. The peeled off nanocomposite film is cut into 4 mm^2^ (2 mm × 2 mm) squares and filled into the vacant positions of the intermediate layer. Finally, the flexible sensor array is assembled by intermediate layer, electrode layers and bumps. The manufacturing methods and fabrication details are described in Supporting Information.

Benefited from the double-sided rough surface structure of the nanocomposite, contact areas in both upper and lower surfaces of nanocomposite films with electrodes change the same and the sensing cell is efficiently deformed under external pressure so as to improve the sensitivity of the device without losing the sensing range. As thermal field-emission scanning electron microscopy (SEM) (Hitachi S-4800, 15 kV) images show in Fig. 2b–g, the rugged structure distributes irregularly on the surface of nanocomposites and has good consistency on the entire scale of two sides’ surfaces (Fig. 2b–d). The measured height distribution data of the surface of nanocomposites by laser scanning confocal microscopy (LSCM) (LSM700, Carl Zeiss Shanghai Co., Ltd) and the obtained probability density distribution of heights are shown in Figs. S2, S3. It is indicated that the probability density distribution of the height of rough surface structure is approximately in accordance with the Gauss distribution with the mean value μ of 39.15 μm and the standard deviation σ of 5.21 μm. Figure S4 shows the piezoresistance of nanocomposites with different surface roughness, which indicates high sensitivity of surface morphology with P800 grit degree and the effect of surface rough structure on increasing sensitivity of nanocomposites in comparison with the planar structure.

A number of micro-caves are formed deeply beneath the surface of flexible nanocomposite after the rough surface structure transferred from the abrasive paper. As shown in Fig. 2e–g, at the surface of micro-caves, CNTs extend out of the nanocomposite matrix disorderly which are circled out by red dashed lines. This reveals the anfractuous and intertwining arrangement of CNTs in the nanocomposite resulting in a large number of intricate conductive networks in the nanocomposites, and the CNTs on the surface of porous structures will contact each other to generate more conductive channels and achieve a higher sensitivity under compressive stress.

The resistance of sensing cells in array is composed of bulk resistance of nanocomposites and contact resistance between nanocomposite surfaces and electrodes. Compared with the planar solid structure, the rough and porous surface structure makes contact areas between the nanocomposites and electrodes decrease and the resistivity increase, so the piezoresistive cell has a larger initial resistance. The smaller contact area and porous surface structure would largely increase the resistance variation range under the same variation extent of pressure compared to bulk resistor. The area change of contact surfaces between nanocomposites and electrodes is the main factor affecting the overall resistance value since the cross-area of piezoresistive nanocomposites is almost unchanged under external pressure compared to the area change of the upper and lower surfaces.

According to the definition of Young’s modulus and the theory of percolation [51], the sensitivity of piezoresistive cells can be written as Eq. 1:

1 $$S = \frac{1}{E} \cdot \left( {1 - \frac{{l \cdot A_{m} }}{A} \cdot f\left( h \right)} \right) + \frac{k}{{\left( {\omega - \omega_{0} } \right)^{\alpha } }}$$

where *E* is effective Young’s modulus of the nanocomposite cell, *l* is the length, *A* is the cross-area, *k* is the correlation coefficient between the content of CNTs and the resistivity of nanocomposites, *ω* is the content of CNTs, $\omega_{0}$ is the threshold content of CNTs just forming the conductive network in nanocomposites, *α* is the factor related to geometrical structure, *A*_*m*_ is the maximum contact area between nanocomposites and electrodes, which is a fixed value and *f(h)* is the probability density function of heights. The detailed derivation process is shown in Supporting Information.

From the analysis above, the sensitivity of the sensor is mainly attributed to the rough and porous surface structure and the content of CNTs in the nanocomposite. The rough porous surface structure determines the effective Young’s modulus of nanocomposite cells, and the content of CNTs influences the density of conductive networks inside the nanocomposite. Therefore, the sensitivity of the device can be modulated effectively by regulating the roughness of surface structure and adjusting the mass fraction of CNTs in the piezoresistive nanocomposite. From Eq. 1, as the pressure increases, the strain of piezoresistive composites becomes larger resulting in a larger effective Young’s modulus. Meanwhile, the number of conductive channels formed by the interconnection of CNTs networks increases. The above two factors contribute to the decrease in the device sensitivity.

## Results and Discussion

### Dynamic Characteristics

**Fig. 3:**
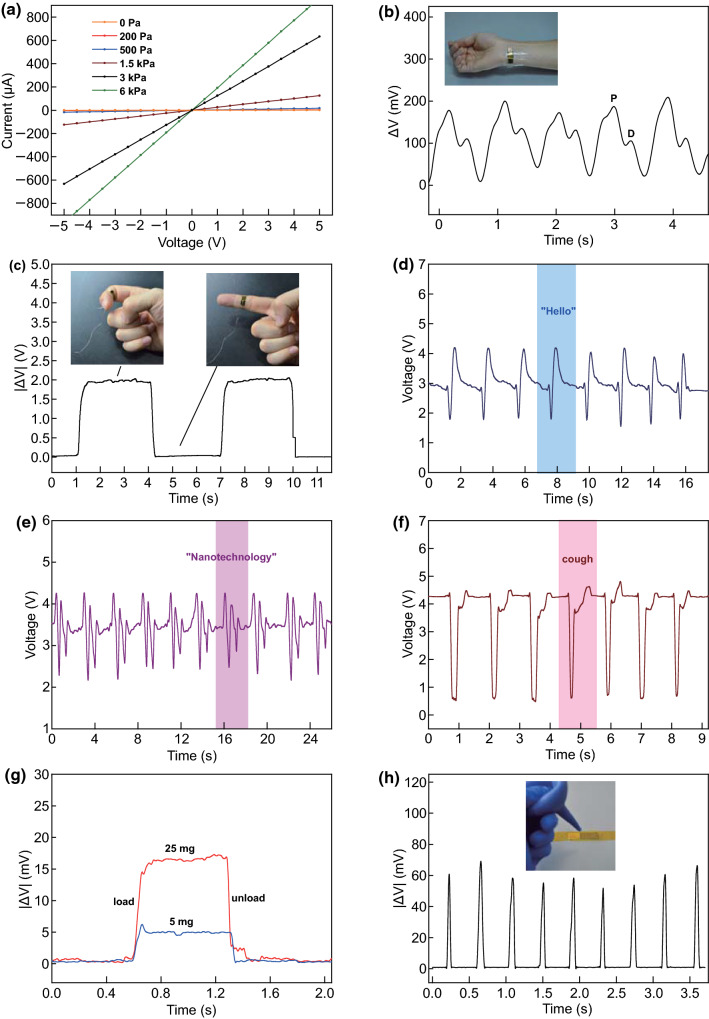
**a** Current–voltage curves of the sensor from − 5 to 5 V under various pressures. **b** Detection of human wrist pulses. **c** Monitoring human finger bending. **d**–**f** Detection of different speech pronunciation and cough vibration. **g** Detection of micro-pressure of 25 mg and 5 mg. **h** Detection of local weak airflow

In order to take full advantage of the excellent mechanical properties of nanocomposites with double-sided rough structure, a single sensor with the same structure and process procedure as the sensor array was fabricated in parallel. The size of piezoresistive sensing unit in the device is 4 mm × 8 mm, and the sensor unit is powered by a 5 V constant voltage source and connected in series with a constant resistance to form a circuit. The signal of voltage change of the sensor unit is collected and displayed by oscilloscope (Keysight DSOX3034T). As illustrated in Fig. 3a, the electrical property of the piezoresistive unit under different normal pressures has been tested. The measured current–voltage curves have good linearity, indicating Ohmic contact characteristic of the device, and the slope of *I*–*V* curves increases with the applied pressure due to the decrease in resistance accordingly.

Figure 3b and Movie 1 (Supporting Information) illustrate the capability of the sensor in detecting human wrist pulses. Two peaks *P* and *D* in each cycle can be clearly identified in the pulse waveform, representing percussion peak and dicrotic wave peak, respectively. Information in wrist pulse waveforms, such as the peak ratio and interval of peak *P* and peak *D*, can be applied to estimate human activities and assists diagnosis of diseases such as pancreatitis and duodenal bulb ulcer [52, 53]. Next, the pressure sensor unit is applied to another application of monitoring various body activities such as finger bending, wrist rotation and arm bending, as shown in Fig. 3c, Fig. S5 and Movie 2. These results imply many potential applications of our tactile sensor in biomedical, human monitoring and disease diagnosis with convenience, flexibility and non-invasiveness ensured. What is more, the single sensor is used to measure the motion of the muscle in throat when a person speaks, as shown in Fig. 3d–f and the insert shows the location. Comparing with finger bending and throat muscle movement in Fig. 3c–e, the compression strain of the sensing unit caused by wrist pulse beating is much smaller and the amplitude of corresponding voltage variation (Fig. 3b) is in the order of hundred mVs because the intensity of pulse beating is much smaller than that of muscle movement. It is clear that the signals measured by sensor show obvious periodicity and consistency when the human body speaks different words and the signal of coughing is sharp and violent, which can be easily identified. Therefore, the device shows good basis for monitoring and recognizing human voice utilizing machine learning methods. Figure 3g shows the detection limit of pressure sensors is 5 mg (≈ 0.5 Pa), and Fig. 3h illustrates the response of the sensor unit to continuous airflow pulses, indicating high sensitivity of the device.

### Piezoresistive Properties of the Electronic Skin Sensor and Applications

**Fig. 4:**
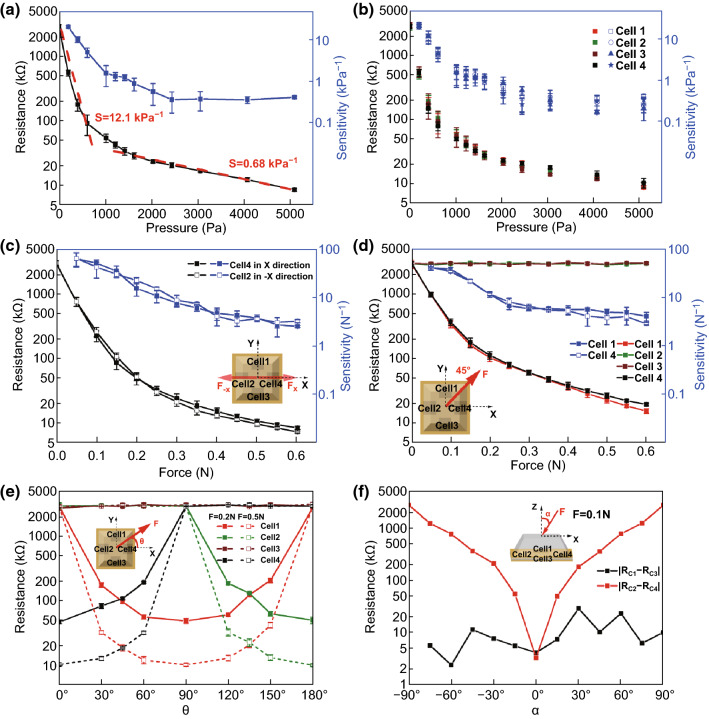
**a** Resistance response and pressure sensitivity of the tactile sensor under normal pressure. The error bars represent standard deviations. **b**, **c** Resistance responses and sensitivities of different cells beneath the bump in one element under normal pressure and tangential forces in *x* direction and − *x* direction. **d** Response curves of cells under tangential forces in 45° direction. **e** Resistance change of each cell to angles in XOY plane (0° ≤ *θ* ≤ 180°), applied forces *F* = 0.2 and 0.5 N. **f** Absolute change of resistance difference of corresponding cells to angles in XOZ plane (− 90° ≤ α ≤ 90°), applied force *F* = 0.1 N. *R*_*C*1_, *R*_*C*2_, *R*_*C*3_ and *R*_*C*4_ represent the resistance of Cell 1, Cell 2, Cell 3 and Cell 4, respectively

The piezoresistive properties of the tactile sensor array were measured by a customized experimental setup, as shown in Fig. S6. The responses of the cells under normal pressures, tangential forces and analysis of decoupling spatial forces are shown in Figs. 4 and S7, S8. Figure 4a demonstrates the resistance response of one cell, and two sensitivity stages can be observed under a wide range of normal pressure. To explain this phenomenon, at first stage, the effective Young’s modulus of the piezoresistive cell is relatively small and the density of conductive networks in nanocomposites is at the minimum condition. According to Eq. 1, lower effective Young’s modulus and looser CNTs networks result in greater sensor sensitivity because smaller effective Young’s modulus leads to a larger deformation of the composite under the unit pressure and a larger deformation causes larger relative variation of resistance. The sensitivity of the tactile sensor reached 20.8 kPa^−1^ in the range of < 200 Pa and 12.1 kPa^−1^ in the regime of < 600 Pa. As the increase in pressure, the contact area between nanocomposites and electrodes and compressive strain of nanocomposites become increasingly larger. At this stage, the effective Young’s modulus is higher and more conductive channels arise in the nanocomposite due to the decrease in the distance between CNTs, leading to the lower sensitivity of the sensor. The sensitivity of the tactile sensor is 0.68 kPa^−1^ as the pressure > 1 kPa and keeps the same within the pressure region of 1–5 kPa. The characteristic of the two stages sensitivity enables our tactile sensor to realize high sensitivity under small pressure as well as to maintain a large measuring range without saturation under high pressure, which is very suitable for multi-occasion applications of robots and human–machine interaction.

Figure 4b shows resistance responses and sensitivities of four cells beneath the bump in one element, and more piezoresistance characterization of cells is shown in Fig. S9. The deviation between different cells is less than 12% implying good piezoresistive consistency. Consequently, the synchronous resistances change of four cells can be employed to infer the applied normal force. Figures 4c, d and S7 illustrate resistance responses and sensitivity curves of the corresponding cells under tangential forces in different directions. We can see that under the tangential force along *x*-axis (Figs. 4c and S7), only the resistance of the cell along the direction of force decreases owing to compression of the cell, whereas the resistances of the other three cells do not change significantly. The sensitivity of tangential force can reach 59.9 N^−1^ in the scope of < 0.05 N and be over 2.3 N^−1^ as the tangential force increases to 0.6 N. When the direction of tangential force rotates to 45° in the XOY plane (Fig. 4d), Cell 1 and Cell 4 are subjected to the same compressive strains and approximate identical resistance changes, while the resistances of Cell 2 and Cell 3 remain nearly unchanged without compressive stress. Universally, tangential forces (0.2 N and 0.5 N) with directional angle of 0° ≤ *θ* ≤ 180° in the XOY plane (Fig. 4e) are applied on the sensing element. At *θ* = 0°, Cell 4 receives the maximum force and the corresponding resistance reaches minimum. As *θ* changes from 0° to 90°, the roles of Cell 1 and Cell 4 swap and Cell 1 reaches its minimum resistance value at *θ* = 90°. The same phenomenon can be observed for Cell 2 and Cell 3 at 90° ≤ *θ* ≤ 180°.

In addition, experiment is conducted out of the XOY plane as well. A 0.1 N force was applied to the bump with different angles (− 90° ≤ α ≤ 90°) to z axis in the XOZ plane, and the absolute values of resistance difference of corresponding cells are displayed in Fig. 4f. The |R_C2_–R_C4_| shows a similar monotonical increase trend as enlarging the angle to *z* axis due to the continuously increasing compressive strain difference between Cell 2 and Cell 4. By contrast, |R_C1_–R_C3_| is no more than 28 kΩ and the approximate axisymmetry of the |R_C2_–R_C4_| curve on α = 90° reflects the consistency among different cells. Therefore, the amplitude and angle of the spatial force in XOZ plane can be obtained via the resistance difference using the |R_C2_–R_C4_| curve, and vice versa. Considering the symmetrical distribution and similar structure of cells, the same conclusion can be drawn in the YOZ plane and XOY plane at 180° ≤ *θ* ≤ 360°. Table 1 displays the comparison of this work with recently reported electronics skin sensors, which indicates the outstanding comprehensive performance such as response time and sensitivity of the proposed 3D tactile sensor.

**Table 1:** Performance of relevant e-skin sensors reported recently

Sensing principle	Normal pressure sensitivity	Normal pressure range	Tangential force sensitivity	Tangential force range	Response time	References
Piezoresistive	1.04 kPa^−1^	20 kPa	–	–	34 ms	[23]
Piezoresistive	1.71 kPa^−1^	5 kPa	2.5 μA/N	1 N	6 ms	[24]
Piezoresistive	35.7 kPa^−1^	5 N	–	–	107 ms	[7]
Piezoresistive	0.05 kPa^−1^	4–100 kPa	–	–	~1 s	[40]
Piezoresistive	2.108%/N	5 N	3.2%/N	0.5 N	–	[54]
Strain gauge	0.676%/N	5 N	0.12%/N	0.5 N	< 0.1 s	[55]
Piezoresistive	12.1 kPa^−1^	6 kPa	59.9 N^−1^	0.6 N	6.8 ms	This work

**Fig. 5:**
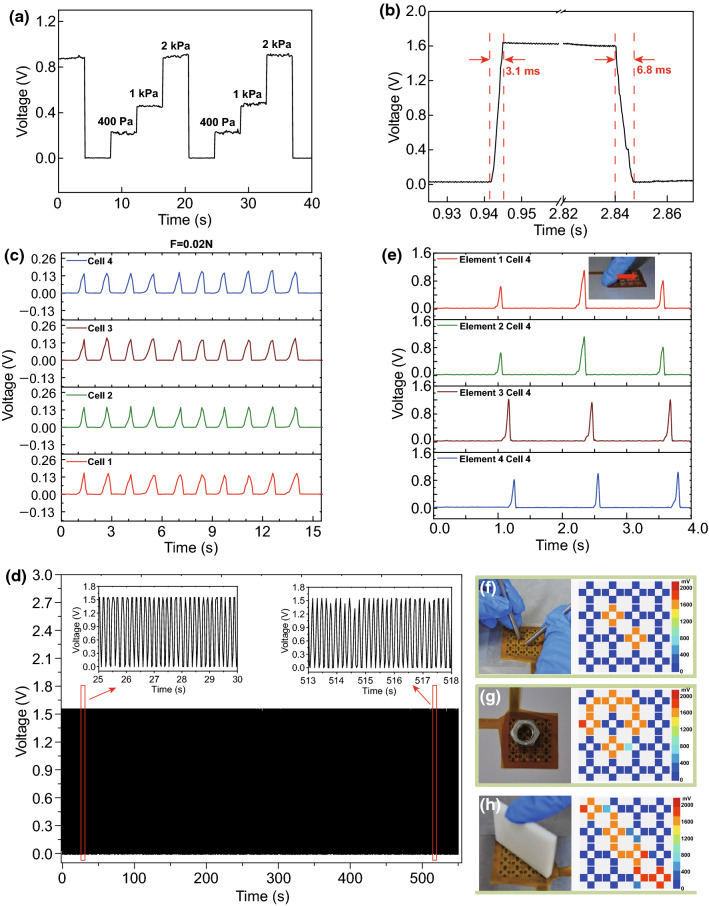
**a** The dynamic voltage response of the sensor under repeated various pressures. **b** Response and recovery time with 5 kPa loading and unloading. **c** Dynamic voltage responses of four cells with continuous loading and unloading of 0.02 N. **d** Good repeatability and durability after continuous about 4000 pressing–releasing cycles. **e** Voltage responses of different cells in the same row under a finger sliding. **f**–**h** Pressure distribution of sensor array with quantitative color under the case of double points pressing, circle pressing and diagonal pressing

The as-prepared tactile sensor device was tested under repeated pressing and releasing cycles in a wide range of compressive deformation. The dynamic response of the device under three repeated pressures (400 Pa, 1 kPa and 2 kPa) is illustrated in Fig. 5a where stable voltage response of the sensor can be seen and a response time measurement was carried out by placing a 0.25 N weight (~ 5 kPa) on the sensor element and removing it quickly. Figure 5b quantifies the response and recovery time under a 5 kPa pressure as 3.1 and 6.8 ms, respectively, implying an ultra-fast dynamic response in several milliseconds order of the sensor even under high pressure.

In order to test the dynamic stability performance of the sensor, 4000 continual human finger pressing–releasing cycles with interval of ~ 0.12 s have been applied to the cell and continuous force loading–unloading cycles with 0.02 N (interval of 1.4 s) and 0.05 N (interval of 1.2 s) have been applied to one element (4 adjacent cells), respectively. Dynamic voltage responses of the sensor are depicted in Fig. 5c, d and Figure S10a, respectively. We can see that the sensor shows good stability, repeatability and consistency, which are important for wearable tactile sensing applications. Also, three cycles of human finger sliding have been applied to the sensor and voltage outputs of different cells (Cell 4, Element 1–4) are plotted in Fig. 5e, from which successive voltage pulses can be seen when the finger glides across the surface of corresponding cells. According to the formula of speed (*ν* = *d*/*t*, *d* is the distance between two cells and *t* is the time interval between two pulses), the speed of a sliding can be derived.

On the basis of sensitive detection for contact force and recognization of contact shape by sensor array, a computer interface is programed to quantify the distribution of pressure applied on the device. Figures 5f–h and S10b–d display the pressure distribution mapping under various strategies of applying force, and the color represents corresponding output voltage of each cell in the sensor array. The distribution of contact forces on the device can be seen intuitively, and the magnitude and direction of the contact force, the shape and moving direction of the contact object can be obtained as well. The corresponding mapping of pressure distribution and the magnitude of forces are obtained when the sensor array is contacted by double points, one circle, diagonal, single point, one row and one column pressing, respectively. It demonstrates the capacity of 3D force sensing by combining pre-calibrated data. For further application demonstration with our tactile sensor, the sensor array is combined with a robotic arm (LW4a, SCHUNK Co.) to grasp objects as exhibited in Fig. S11a, c. The empty plastic bottle and the bottle containing 400 mL pigmented water are clamped stably, respectively, without obvious deformation, and the color array indicating the force distribution on sensor array is shown in Fig. S11b, d. Obvious tangential force and normal pressure distribution on the sensor array can be seen due to the gravity of water. By contrast, for the empty bottle, only slight distributed normal pressure can be seen in the contact area because of its small mass. In addition, output voltages variation of different cells during a grasping process is illustrated in Fig. S12. The real-time 3D force distribution data can contribute to the robotic application of grasping objects without damage by controlling the force and angles of the robotic arms through the machine learning algorithm.

## Conclusions

In summary, a 4 × 4 flexible tactile sensor electronic skin based on special double-sided rough porous structure of CNTs-PDMS piezoresistive nanocomposites is developed for 3D contact force detection. The excellent physical and electrical properties of CNTs network enable sensor elements to maintain stability and good electrical conductivity under compressive stress. The double-sided rough microstructure dominates the ultra-high sensitivity and detection resolution of the sensor. Various experiments have been designed and established to quantify the piezoresistive characteristics and dynamic properties of the device under several tactile sensing applications. Experimental results show strong relationship between cell resistances and applied forces, and each axis force component can be calculated by combining resistances of four cells in one element and the pre-calibrated data. The sensitivity of the tactile sensor is 12.1 kPa^−1^ in the range of < 600 Pa and 0.68 kPa^−1^ in the regime exceeding 1 kPa for normal pressure. The sensitivity of tangential force reaches 59.9 N^−1^ in the scope of < 0.05 N and is over than 2 N^−1^ in the region of < 0.6 N, which is the highest within the scope of our knowledge. With the flexible materials and simple fabrication process, the sensor array is able to manufacture in low cost with good repeatability, stability and uniformity and the response time of the tactile sensor is down to several milliseconds under high pressure. The sliding direction on surface can be detected effectively, and the sliding speed is able to derive. In addition, the sensitive single sensor has also been successfully demonstrated to measure wrist pulses and human limbs movements, implying potential applications of the tactile sensor in gentle touch measuring, human monitoring, biomedical and disease diagnosis. Finally, the sensor array is combined with robotic arm to grasp objects with real-time pressure distribution mapping, implying the capacity of applications in integrated robots.

## Electronic supplementary material

Below is the link to the electronic supplementary material.

Supplementary material 1 (PDF 874 kb)

Supplementary material 2 (AVI 2942 kb)

Supplementary material 3 (AVI 5969 kb)

Supplementary material 4 (AVI 1790 kb)
