# Supplementary material for: Flexible Tactile Electronic Skin Sensor with 3D Force Detection Based on Porous CNTs/PDMS Nanocomposites
Source: Nanomicro Lett. 2019 Jul 16;11:57. doi: 10.1007/s40820-019-0288-7 (PMC7770749; doi:10.1007/s40820-019-0288-7)
Supplement: Supplementary file 1 — Supplementary material 1 (PDF 874 kb) [file 40820_2019_288_MOESM1_ESM.pdf]

Supporting Information for

Flexible Tactile Electronic Skin Sensor with 3D Force Detection based on Porous CNTs/PDMS Nanocomposites

Xuguang Sun[1, 2, ‡], Jianhai Sun[1, ‡], Tong Li[1], Shuaikang Zheng[1, 2], Chunkai Wang[1, 2], Wenshuo Tan[1, 2], Jingong Zhang[3], Chang Liu[1], Tianjun Ma[1], Zhimei Qi[1], Chunxiu Liu[1, *], Ning Xue[1, *]

[1]State Key Laboratory of Transducer Technology, Institute of Electronics Chinese Academy of Sciences (IECAS), Beijing 100190, People's Republic of China

[2]School of Electronic, Electrical, and Communication Engineering, University of Chinese Academy of Sciences (UCAS), Beijing 100049, People's Republic of China

[3]School of Microelectronics, University of Science and Technology of China (USTC), Hefei 230026, People's Republic of China

[‡]X.Sun and J.Sun contributed equally to this work.

*Corresponding authors. E-mail: xuening@mail.ie.ac.cn (Ning Xue); cxliu@mail.ie.ac.cn (Chunxiu Liu)

S1 Methods

S1.1 Fabrication of Nanocomposites

The MWNTs/PDMS (Sylgard 184, DowCorning Co, USA) nanocomposite was produced by solution mixture and solvent evaporation because the agglomeration of MWNTs would occur if MWNTs were dispersed into the PDMS matrix directly. In this work, chloroform was chosen as the solvent for blending MWNTs with PDMS uniformly. 6 wt% of hydroxyl modified MWNTs (TNMH1, Outer Diameter: < 8 nm, Length: 10−30 μm, Purity > 98%, −OH 5.58 wt%, Chengdu Organic Chemicals Co. Ltd., Chinese Academy of Sciences, China) was poured in chloroform solvent and phenylmethylsiloxane (PPMS) was added into the solution at a mass ratio of 1:10 (PPMS: PDMS) to actualize better dispersibility in PDMS matrix. Then the solution was treated by ultrasonic water bath for 1 h at 30 °C to make MWNTs homogeneously mixed with the chloroform solvent. Synchronously, the PDMS was poured into the chloroform solvent at a mass ratio of about 10% (PDMS/ chloroform) and the solution was stirred for 5 minutes using a magnetic stirrer. After that, the chloroform solution of MWNTs and the chloroform solution of PDMS were mixed up together and sonicated in an ultrasonic bath at 70 °C till the chloroform solvent was completely evaporated. Then the curing agent (1/10 of the mass of PDMS) was added into MWNTs-PDMS nanocomposite with full agitation. After degassing in vacuum in a vacuum drying oven for 10 minutes, the MWNTs-PDMS nanocomposite was completed.

S1.2 Fabrication of Microstructured PDMS Intermediate Layer and Nanocomposites

A 1-μm-thick layer of parylene-C was deposited on the rough surface of the abrasive paper mould (grit designation: P800, Buehler Co, USA) by vacuum deposition system (PDS2010, Specialty Coating Systems Inc. USA) and a 200-μm-thick PDMS was spin coated (600 rpm, 30 s) on a 5 inches mould substrate and thermally cured at 120 °C for 30 min in the hot drying cabinet. After peeling off from the mould, the PDMS film was deposited with 1-μm-thick layer of parylene-C. Similar to the process of manufacturing PDMS intermediate layer, the prepared MWNTs-PDMS nanocomposite was knife coated on the same mould with parylene-C deposition using hard mask and then the flexible PDMS film deposited with parylene was covered on the surface of the nanocomposite with the rough surface downward. The thickness of the nanocomposite film can be adjusted by changing the thickness of the hard mask. After heating at 120 °C for 30 minutes in vacuum, the PDMS film was peeled off and the nanocomposite film possesses double-sided rough surface structure.

S1.3 Fabrication of the Flexible Tactile Sensor Array

The intermediate isolating PDMS layer was stacked on the lower electrode layer which is a 20-μm-thick flexible printed circuit (FPC) board with pre-patterned electrodes and electrical wires. Nanocomposites cells were filled into indentations of intermediate PDMS layer. Then the upper electrode layer was attached on the intermediate PDMS layer using small amount of PDMS and the device was heated at 50 °C for 2 h to solidify the PDMS. The external force with various sizes and directions were applied by a force meter and three-axis stage (illustrated in Fig. S4).

S2 Scanning Circuit System

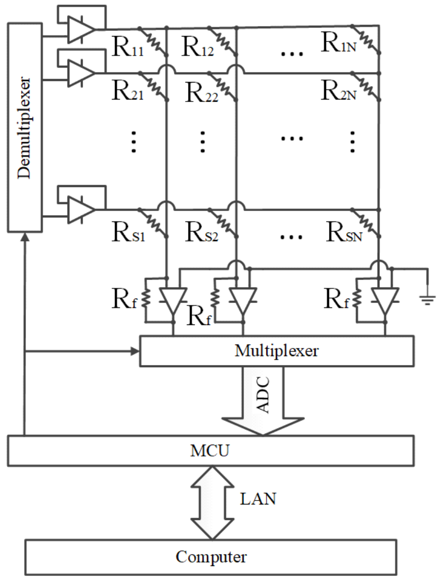

Fig. S1 The schematic diagram of the array scanning circuit system

As shown in Fig. S1, the array scanning system consists of scanning circuit, multi−control unit (MCU) and computer interface. In the scanning circuit, a row of standard resistances marked as R_{S1} to R_{SN} is added for initial calibration. The resistances from piezoresistive nanocomposite cells array marked as R_{ij} (i is the row number and j is the column number) are connected to the circuit. Each row and column has an operational amplifier (OA) connected to resistors. When a row of OA is selected by MCU and high voltage is applied to its forward input, OAs in other rows are connected to zero potential. The resistance of each piezoresistive nanocomposite cell in the selected row is obtained by measuring the output voltage of each column OA, separately. Followed by digitalization through analog−to−digital converter, the data was transmitted to computer through LAN. The relationship between output voltage and resistance is as Eq. S1:

$$V_{outij} = -\frac{V_d}{R_{ij}} R_f \tag{S1}$$

Where V_{otuij} is the output voltage of the OA in the j column, V_d is the input voltage of the OA in the i row and R_f is the reference resistance of the OA. Similar to piezoresistive cells, the output voltage of standard resistances can be measured as Eq. S2:

$$V_{outsj} = -\frac{V_d}{R_{Sj}} R_f \tag{S2}$$

And Eq. S3 can be obtained from Eqs. S1 and S2 as:

$$R_{ij} = \frac{V_{outsj}}{V_{outij}} R_{Sj} \tag{S3}$$

From Eq. S3, we can see that the input voltage V_d has no effect on the calculation of the resistance of one cell in the sensor array. So the scanning circuit can effectively avoid the influence of the fluctuation of input voltage on the resistance value and improve the measurement accuracy. What's more, row−by−row scanning can effectively avoid crosstalk between different rows and columns.

S3 LSCM Height Distribution Mapping

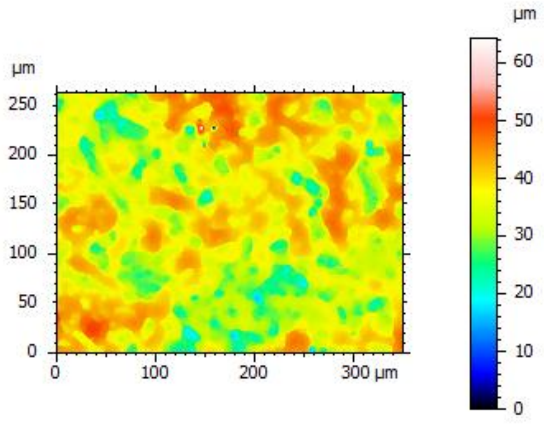

Fig. S2 LSCM height distribution mapping of selected nanocomposite surface area

S4 Probability Distribution Results

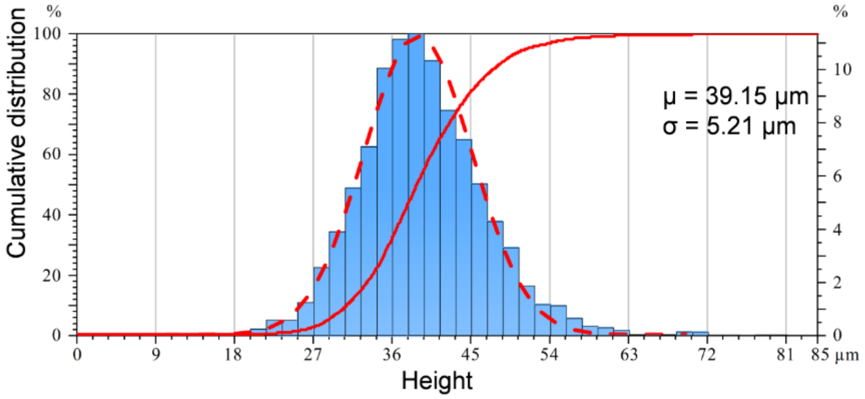

Fig. S3 Cumulative distribution and probability density of the height of nanocomposites surface structure

S5 Piezoresistive Characteristics of Different Surface Roughness

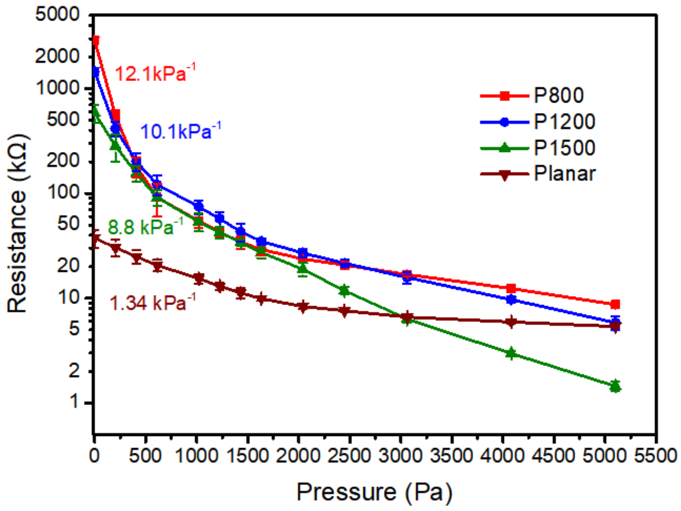

Fig. S4 Piezoresistive curves of sensing nanocomposites with different surface roughness

Figure S4 shows the comparison of piezoresistance of sensing nanocomposites with different surface roughness. And the sensitivity of different surface structures in the range of < 0.6 kPa is displayed in the figure.

S6 Detailed Derivation Process of Sensitivity

The resistance of piezoresistive cells in tactile sensor array is composed of bulk resistance of nanocomposites and contact resistance between nanocomposite surfaces and electrodes. Resistance is defined as Eq. S4:

$$R = \frac{\rho \cdot l}{A} \qquad \text{(S4)}$$

where R is the resistance, l is the length, A is the cross area and ρ is resistivity of the nanocomposite.

According to the formula of resistance and the variation of resistance, the sensitivity of piezoresistive cells can be written as Eq. S5:

$$S = \frac{|\Delta\rho/\rho|}{\Delta P} + \frac{|\Delta l/l|}{\Delta P} - \frac{\left|\frac{\Delta A}{A}\right|}{\Delta P} \qquad \text{(S5)}$$

where S is the sensitivity, ΔP, $\Delta\rho$ and Δl are the variation of pressure, resistivity and length respectively. ΔA can be considered as the area change of contact surfaces between nanocomposite and electrodse since the cross area of piezoresistive nanocomposites is almost unchanged under external pressure compared to the area change of the upper and lower surfaces.

According to the fomula of resistance, the variation of resistance can be written as Eq. S6:

$$\Delta R = \frac{\Delta\rho \cdot l}{A} + \frac{\Delta l \cdot \rho}{A} - \frac{\Delta A \cdot \rho \cdot l}{A^2} \qquad \text{(S6)}$$

Sensitivity is defined as Eq. S7:

$$S = \left|\frac{\frac{\Delta R}{R}}{\Delta P}\right| \qquad \text{(S7)}$$

Merge Eqs. S6 and S7, the sensitivity is:

$$S = \frac{|\Delta\rho|/\rho}{\Delta P} + \frac{|\Delta l|/l}{\Delta P} - \frac{|\Delta A|/A}{\Delta P} \qquad \text{(S8)}$$

According to the theory of percolation, the relationship between the content of MWNTs and the resistivity of nanocomposites can be written as Eq. S9:

$$\frac{|\Delta\rho|/\rho}{\Delta P} = k \cdot \frac{1}{(\omega - \omega_0)^{\alpha}} \qquad \text{(S9)}$$

where k is the correlation coefficient between the content of MWNTs and the resistivity of nanocomposites, ω is the content of MWNTs, ω_0 is the threshold content of MWNTs to just form the conductive network in nanocomposites, α is the factor related to geometrical structure. According to the definition of Young's modulus, where $E = \Delta P/(\Delta l/l)$, the second term of Equation S8 can be expressed as $1/E$.

According to the measured data of the probability density of height distribution, the area proportion distribution of different heights basically conforms to the Gauss distribution. And the contact area between nanocomposites and electrodes at different heights is approximately equal to the product of cumulative probability at this height and maximum possible contact area. So the small change of contact area can be approximately equal to the product of probability density at a

certain height and maximum contact area. Because the rough structure is distributed on the surface of the nanocomposite only, the maximum contact area can be regarded as the area of the nanocomposite cell. So the last term of Eq. S8 can be written as:

$$\frac{\frac{|\Delta A|}{A}}{\Delta P} = \frac{\left|\frac{\Delta A}{\Delta l}\right|}{A \cdot \frac{\Delta P}{\Delta l}} = \frac{\left|\frac{\Delta A}{\Delta l}\right|}{A \cdot \frac{E}{l}} = \frac{l}{A \cdot E} \cdot f(h) \cdot A_m \text{ B} \qquad \text{(S10)}$$

Where A_m is the maximum contact area between nanocomposites and electrodes, which is a fixed value and $f(h)$ is the probability density function of heights. The compressive strain and the area change caused by pressure on upper and lower surfaces can be regarded same due to the double–sided structure of nanocomposites. The relationship between the height and length variation of nanocomposites and the probability density function of heights can be written as:

$$h = \frac{l_0 - l}{2}, f(h) = \frac{1}{\sqrt{2\pi}\sigma} exp\left(-\frac{(h-\mu)^2}{2\sigma^2}\right) \qquad \text{(S11)}$$

where l_0 is the initial length of nanocomposites. Thus according to Eqs. S8, S9 and S10, the sensitivity can be expressed as:

$$S = \frac{1}{E} \cdot \left(1 - \frac{l \cdot A_m}{A} \cdot f(h)\right) + \frac{k}{(\omega - \omega_0)^{\alpha}} \qquad \text{(S12)}$$

S7 Applications of the Single Sensor

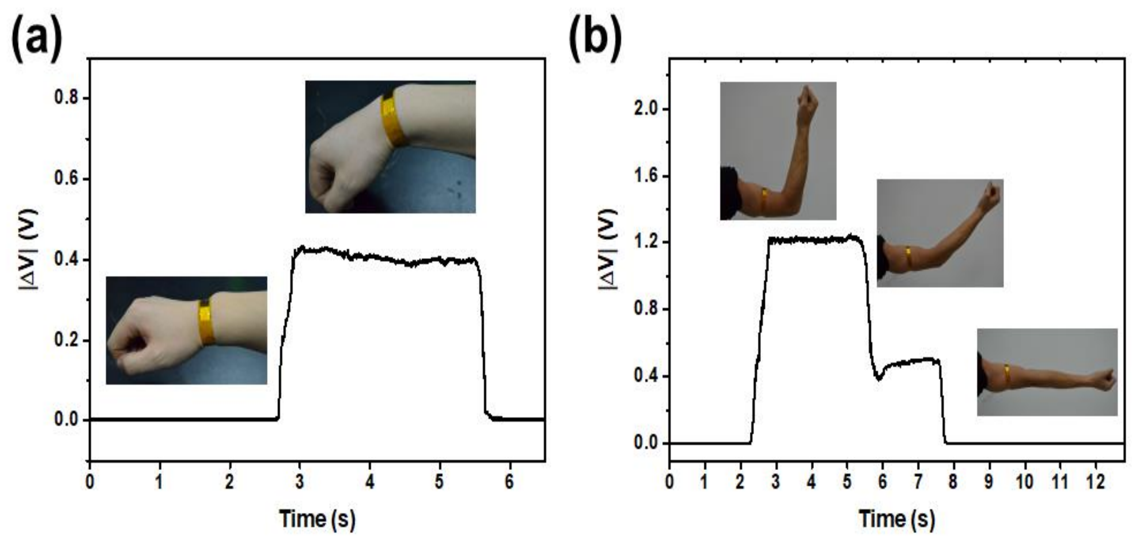

Fig. S5 a Detection of wrist bending. **b** Detection of arm bending

S8 Experimental Measuring System

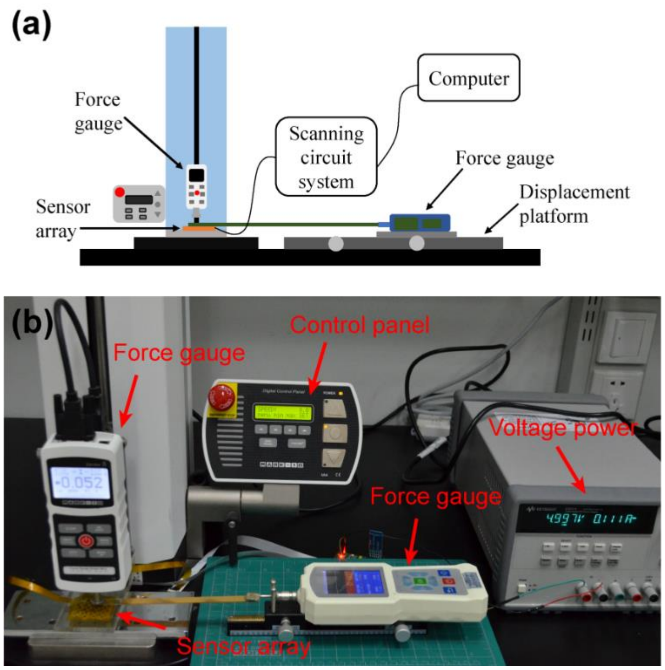

Fig. S6 a Schematic sketch and **b** optical photograph of experimental system for measuring the piezoresistive properties of tactile sensor

As shown in Fig. S6, the customized experimental measuring system is composed of a three−axis platform and two force gauges (M3, Mark−10 Co, USA, with accuracy of 2 mN) which can apply normal force and tangential force to the sensor array. The pure tangential force is provided by the force gauge placed horizontally and the horizontal thin narrow trip at the front of the force gauge is stuck to the bump upper surface. When the pressure gauge moves along the displacement guide trail, the bump is subjected to the static friction force which can be equivalent to the pure tangential force.

S9 Resistance Changes of Different Cells under Tangential Force

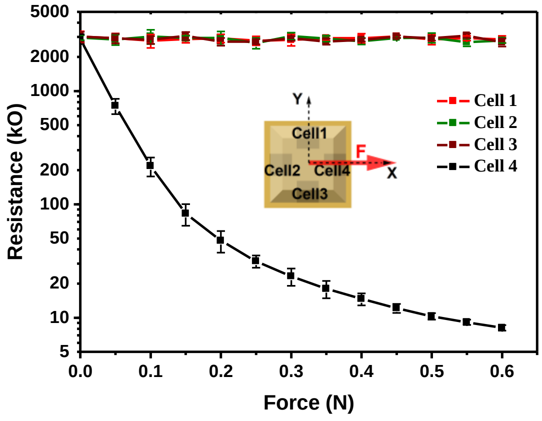

Fig. S7 Resistance changes of different cells in one element under tangential force in x–axis

S10 Decoupling Analysis of Spatial Force

For any spatial force, it can be decomposed into components along the three axes of X, Y, and Z, which is shown in Fig. S8a. Considering the distribution of four piezoresistive cells in the sensor element, the compressive deformation of each cell under normal pressure can be regarded as equal to the displacement of the bump of the element (d_Z).

$$d_z = d_1 = d_2 = d_3 = d_4 \tag{S13}$$

Therefore, the normal force can be calculated by

$$\mathrm{F}_z = k_z d_z = k_z \frac{d_1+d_2+d_3+d_4}{4} \tag{S14}$$

where k_z is the spring constant in Z-axis direction.

And when the tangential force in the X-axis direction is applied to frustum bump, a torque around the Y-axis will be caused on the element under the action of the force, which is shown in Fig. S8c. The expression of the torque can be given as

$$\tau_x = k_x \alpha = k_x \frac{(d_2-d_4)/2}{L/2} = F_{x\prime} \times h = F_x \cos\alpha \times h \approx F_x \times h \tag{S15}$$

where k_x is the spring constant in X-axis direction, α and L are rotation angle and side length of the bump respectively. The rotation angle caused by tangential force is pretty small in the case of tactile contact, so the cosine value of α can be approximately considered as 1. Thus the tangential force in X-direction can be given as:

$$F_x = k_x \frac{(d_2-d_4)}{Lh}, \quad d_2 = -d_4 \tag{S16}$$

Analogously, the tangential force in Y-direction can be given as:

$$F_y = k_y \frac{(d_3-d_1)}{Lh}, \quad d_3 = -d_1 \tag{S17}$$

According to the relationship among the spatial force, three components of the force and angles between projection of the force and corresponding planes, the three-dimensional spatial force can be synthesized.

$$F = \frac{F_z}{\cos\alpha} = \frac{F_x}{\sin\alpha\cos\theta} = \frac{F_y}{\sin\alpha\sin\theta} \tag{S18}$$

According to the above analysis, any spatial force can be obtained according to the deformation of four cells in the sensor element and vice versa.

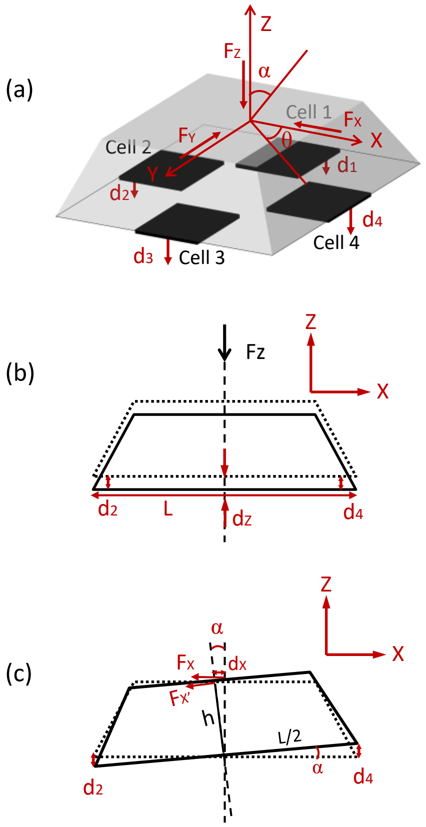

Fig. S8 a Simplified structure model of a sensor element with three-dimensional spatial coordinates. **b**, **c** Simplified movement diagrams of cross section of a bump under a normal force and a tangential force in X-direction

S11 Uniformity of the Sensor Array

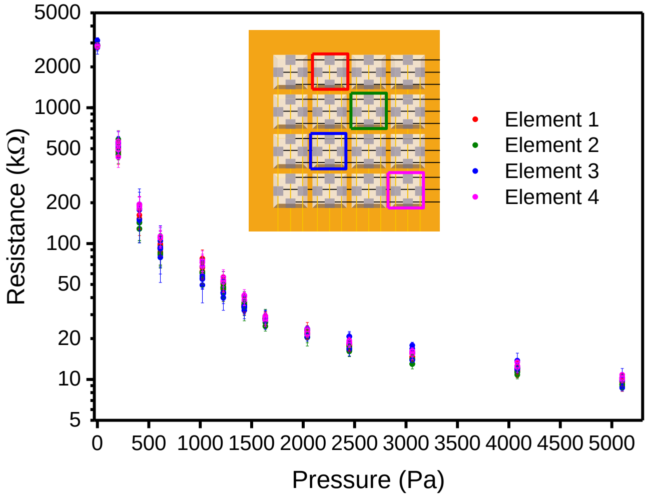

Fig. S9 Piezoresistive characterization of cells in different elements of the sensor array

S12 Dynamic Responses of the Sensor

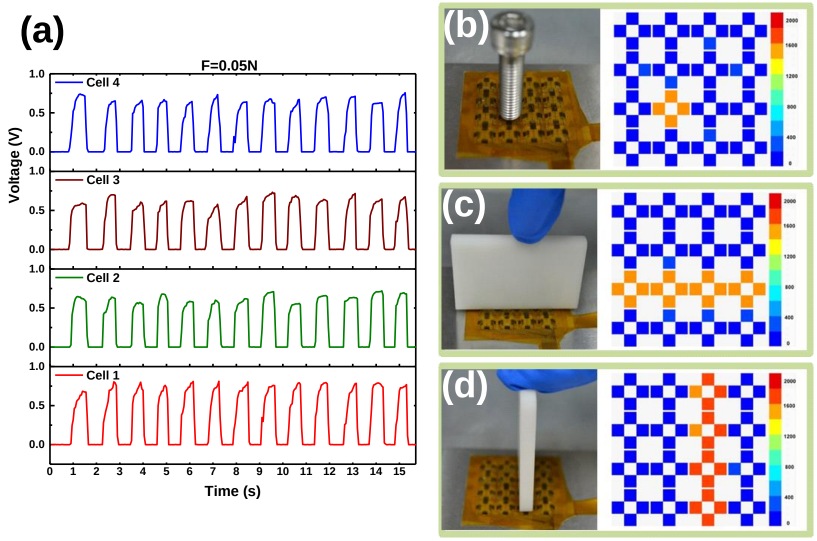

Fig. S10 a Dynamic voltage responses of four cells with continuous loading and unloading of 0.05 N. **b–d** Pressure distribution of sensor array with quantitative color under the cases of single point pressing, one row pressing and one column pressing respectively

S13 Combination of the Sensor Array with a Robotic Arm

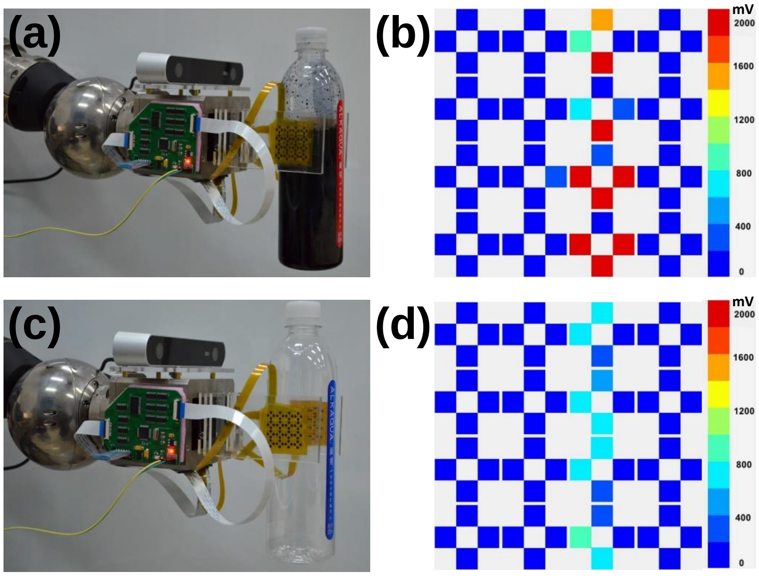

Figure S11. Application of Grabbing Objects by combining the sensor array and a robotic arm. **a** Tactile sensor array is used for robotic arm to grab a bottle containing 400 mL pigmented water. **b** Pressure distribution of sensor array with quantitative color in **a**. **c** Tactile sensor array is used for robotic arm to grab an empty bottle. **d** Pressure distribution of sensor array with quantitative color in **c**.

As shown in Fig. S11a, c, the sensor array is tightly attached to the transparent acrylic clamp with transparent tape and the clamp is assembled to the end joint of the manipulator controlled by the motor. The array scanning circuit and the wireless transceiver are attached to the side of the end joint with insulation treatment. The two acrylic clamps move towards each other under the drive of the motor to grasp object placed on the plane until the sensor array detects enough contact pressure during the grabbing process. Then the grabbed object is moved to the desired position by the robotic arm under controlling the joint rotation of the manipulator.

S14 Output Voltages during a Grasping Process

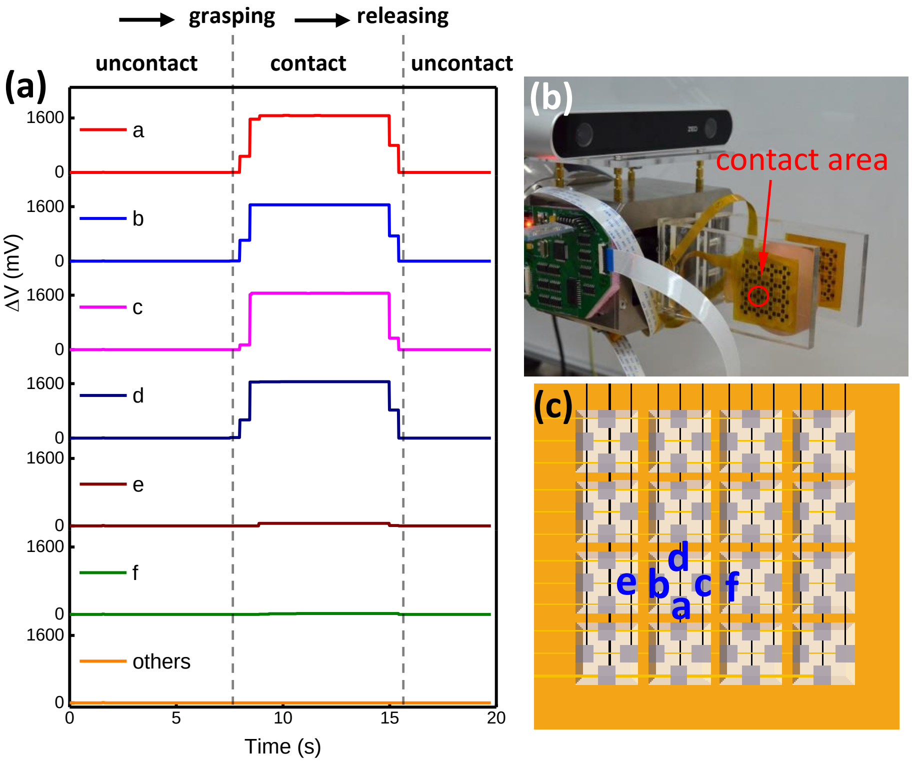

Fig. S12 a Output voltages of different cells during the grasping process. **b** Optical photographs of grabbing an egg. **c** Schematic map of the location of cells

As shown in Fig. S12, the contact area between the egg ellipsoid and the sensor array is relatively small when it is grasped because of the ellipsoid shape of the egg. The variation of output voltages of cell a, b, c, d in contact area can reflect the main stress area in egg grabbing while the voltages of cell e, f change slightly due to a small amount of contact.
